# The Implicated Roles of Cell Adhesion Molecule 1 (*CADM1*) Gene and Altered Prefrontal Neuronal Activity in Attention-Deficit/Hyperactivity Disorder: A “Gene–Brain–Behavior Relationship”?

**DOI:** 10.3389/fgene.2019.00882

**Published:** 2019-09-26

**Authors:** Jiali Jin, Lu Liu, Wai Chen, Qian Gao, Haimei Li, Yufeng Wang, Qiujin Qian

**Affiliations:** ^1^Department of Child Psychiatry, Peking University Sixth Hospital/Institute of Mental Health, Beijing, China; ^2^National Clinical Research Center for Mental Disorders & the Key Laboratory of Mental Health, Ministry of Health (Peking University), Beijing, China; ^3^Centre & Discipline of Child and Adolescent Psychiatry, and Psychotherapy, School of Medicine, Division of Paediatrics and Child Health & Division of Psychiatry and Clinical Neurosciences, The University of Western Australia, Perth, WA, Australia; ^4^Complex Attention and Hyperactivity Disorders Service (CAHDS), Specialised Child and Adolescent Mental Health Services of Health in Western Australia, Perth, WA, Australia

**Keywords:** attention-deficit/hyperactivity disorder, *CADM1*, executive function, imaging genetics, prefrontal cortex, mean amplitude of low-frequency fluctuation

## Abstract

**Background:** Genes related to cell adhesion pathway have been implicated in the genetic architecture of attention-deficit/hyperactivity disorder (ADHD). Cell adhesion molecule 1, encoded by *CADM1* gene, is a protein which facilitates cell adhesion, highly expressed in the human prefrontal lobe. This study aimed to evaluate the association of *CADM1* genotype with ADHD, executive function, and regional brain functions.

**Methods:** The genotype data of 10-tag single nucleotide polymorphisms of *CADM1* for 1,040 children and adolescents with ADHD and 963 controls were used for case–control association analyses. Stroop color–word interference test, Rey–Osterrieth complex figure test, and trail making test were conducted to assess “inhibition,” “working memory,” and “set-shifting,” respectively. A subsample (35 ADHD versus 56 controls) participated in the nested imaging genetic study. Resting-state functional magnetic resonance images were acquired, and the mean amplitude of low-frequency fluctuations (mALFF) were captured.

**Results:** Nominal significant genotypic effect of rs10891819 in “ADHD-alone” subgroup was detected (*P* = 0.008) with TT genotype as protective. The results did not survive multiple testing correction. No direct genetic effect was found for performance on executive function tasks. In the imaging genetic study for the “ADHD-whole” sample, rs10891819 genotype was significantly associated with altered mALFF in the right superior frontal gyrus (rSFG, peak *t* = 3.85, corrected *P* < 0.05). Specifically, the mALFFs in T-allele carriers were consistently higher than GG carriers in ADHD and control groups. Endophenotypic correlation analyses indicated a significant negative correlation between “word interference time” in Stroop (shorter “word interference time” indexing better inhibitory function) and mALFF in the rSFG (*r* = -0.29, *P* = 0.006). Finally, mediation analysis confirmed significant indirect effects from “rs10891819 genotype (T-allele carriers)” *via* “mALFF (rSFG)” to “inhibition (“word interference time”)” (Sobel*z* = -2.47; B = -2.61, 95% confidence interval -0.48 to -4.72; *P* = 0.009).

**Conclusions:** Our study offered preliminary evidence to implicate the roles of *CADM1* in relation to prefrontal brain activities, inhibition function, and ADHD, indicating a potential “gene–brain–behavior” relationship of the *CADM1 gene*. Future studies with larger samples may specifically test these hypotheses generated by our exploratory findings.

## Introduction

Attention-deficit/hyperactivity disorder (ADHD)—characterized by developmentally inappropriate levels of inattention, hyperactivity, and impulsivity—is one of the most common childhood neurodevelopmental disorders with an estimated worldwide prevalence of 5% (American Psychiatric Association, 2013). It is a condition of genetic etiology, with a heritability estimated around 74% (Faraone and Larsson, 2019). To date, a large-scale genome-wide association meta-analysis based on categorical clinical diagnoses—which are derived from observed patterns of symptom clustering—has identified 12 significant loci involved in the underlying biology of ADHD (Demontis et al., 2019). Interestingly, these significant loci do not coincide with those reported by previous candidate genes studies, underscoring the limitations of the candidate gene approach. The Research Domain Criteria (RDoC), proposed by the National Institute of Mental Health, offer a different theoretical framework to re-orientate research approach, in particular, redirect the primary focus from diagnostic categories of ADHD to the functioning of specific domains (i.e., along the continuum from genes to molecules, cells, brain circuitry, cognitive endophenotypes, and behaviors) that are presumed to underlie the clinical manifestations (Musser and Raiker, 2019). In addition to the candidate association approach, this present study also attempts to apply the RDoC approach to explore different investigative avenues to detect associations between genes (putative functional molecules), brain activities, cognitive endophenotypes, and ADHD behaviors, within the context of cell adhesion molecule 1 (*CADM1*) gene.

By topological and functional analyses, a recent study identified potential roles of genes related to cell adhesion pathway, as being implicated in the genetic architecture of ADHD (Lima et al., 2016). In particular, cell adhesion molecule 1 (CADM1), encoded by *CADM1* gene, is a member of the immunoglobulin superfamily with cell adhesion properties, which promote axonal growth, neuronal migration, pathfinding, and synaptic formation in the developing nervous system and is also involved in the formation of neural networks (Fujita et al., 2005; Perez et al., 2015). In central and peripheral nerve system, CADM/SynCAM is essential for myelination *via* moderating adhesion of gliocyte and axon (Niederkofler et al., 2010) and plays pivotal roles in developing neurons by shaping their migrating growth cones and adhesive differentiation in their axo-dendritic contacts (Stagi et al., 2010; Yamagata et al., 2018). An animal model demonstrates in mature neurons the additional function of CADM1 protein molecule, which participates in regulating neuronal plasticity and synapse number (Robbins et al., 2010). Overall, the earlier evidence suggested that CADM1 plays multiple critical roles in maintaining neuronal integrity and functions from development to maturity.

Genes involved in CADM1-related pathways have been implicated in ADHD, such as *ITGA1* or *CDH13* genes related to cell adhesion (Liu et al., 2017) and cell-to-cell communication functions (Hawi et al., 2015). Despite lacking direct evidence from human studies, a recent rodent model reported a relationship between *CADM1* function and ADHD-like behaviors. Altered expression of *CADM1* gene was associated with abnormal diurnal spontaneous activity in mice. When compared with wild type, mice with GFAP-DNSynCAM1 (i.e., dominant negative mutation) displayed increased daytime activity, decreased rest, and nocturnal hyperactivity, and strikingly, these anomalies were reversed by amphetamine. Moreover, higher levels of impulsive and aggressive behaviors were also present, such as jumping out of their cages and attacking other mice, i.e., behaviors consistent with other rodent models of ADHD (Sandau et al., 2012).

Furthermore, two missense mutations of *CADM1* gene—C739A(H246N) and A755C(Y251S)—were found in autism spectrum disorder probands and their family members (Zhiling et al., 2008)—a neurodevelopmental disorder sharing high rates of comorbidity with ADHD (Sharma et al., 2018) and potential common molecular genetic etiologies (Gonzalez-Mantilla et al., 2016). At the molecular level, the mutant variants of *CADM1* gene were associated with abnormal expression of matured oligosaccharide, cell surface trafficking defection, and greater susceptibility to cleavage or degradation (Zhiling et al., 2008). At the cellular level, the mutant *CADM1* gene was also associated with morphological and functional alterations in neurons, including shorter dendrites, impaired synaptogenesis (Fujita et al., 2010), and disruption in protein distribution (Muhle et al., 2004). Compared with normal cells, the abnormal CADM1 proteins in mutant cells accumulated mainly in the endoplasmic reticulum (ER) and induced upregulation of the ER stress marker, known as “C/EBL-homologous protein” (Fujita et al., 2010). Long-term exposure to excessive ER stress can lead to neuronal death, thereby implicating *CADM1* genetic mutation as potentially relevant to aberrant neurodevelopment and related pathogenesis (Muhle et al., 2004)

Interestingly, CADM1 is highly expressed in many brain areas, including the cingulate cortex, parietal lobe, temporal lobe, occipital lobe, amygdala, caudate nucleus, cerebellum, and, especially, prefrontal cortex (http://biogps.org/#goto=genereport&amp;id=23705), all of which represent the most relevant common brain regions identified by neuroimaging studies of ADHD (Liston et al., 2011). Notably, functional magnetic resonance imaging (fMRI) studies highlighted a complex neural network (comprising of prefrontal lobe, parietal lobe, cingulate cortex, cerebellum, and basal ganglia) involved in information processing relevant to ADHD deficits, such as attention, shifting, planning, reward, working memory, and response inhibition (Liston et al., 2011; Carlen, 2017). More specifically, ADHD participants showed reduced inhibitory control associated with lower brain activation of bilateral ventral lateral prefrontal cortex when compared with controls (Norman et al., 2016). Task-specific fMRI study also found differences in ADHD participants: significantly decreased activation in the right inferior frontal cortex while performing inhibition tasks (Hart et al., 2013); increased activation in the left dorsolateral prefrontal cortex while completing working memory tasks (Bedard et al., 2014); as well as under-activation in bilateral inferior prefrontal cortices during visual–spatial switching tasks (Rubia et al., 2010). In other words, certain brain regions with high CADM1 expression overlapped with those related to ADHD and associated neurocognitive deficits. However, no reports have explored the specific genetic effects of *CADM1* polymorphism on these brain structures and/or functions in the context of ADHD.

Intriguingly, despite different strands of compelling evidence on the implicated roles of *CADM1* gene in neurodevelopmental disruption (and likely symptom expression) and potential relevance to ADHD, no genetic variants of *CADM1* have achieved the postulated genome-wide significance (*P* < 10^-8^) or been represented among the genome-wide association studies (GWAS) top hits—in either the meta-analysis or primary GWAS of ADHD (Demontis et al., 2019; Faraone and Larsson, 2019). These findings suggested that the association between *CADM1* genotype and ADHD phenotypes, if it exists, may not be readily detected by conventional genetic analyses. The postulated links between *CADM1* and ADHD may therefore need to be probed by an alternative strategy as guided by the RDoC initiative (which redirects focus on the “gene–brain–behavior” relationships along the continuum of interlinking domains from genes, cells, anatomical regions, functions, and behaviors), instead of conventional diagnostic phenotypes. As an exploratory and hypotheses-generating study, we therefore considered it best to interrogate brain circuitry anomalies by applying an atheoretical probe along the domain continuum. In contrast to task-related fMRI, resting-state fMRI (rs-fMRI) permits evaluation of brain activities without prior theoretical assumptions, representing a more appropriate method to capture spontaneous regional neural activity as indexed by mean amplitude of low-frequency fluctuation (mALFF).

There were two key objectives of this study. First was to examine the association of *CADM1* gene in relation to ADHD psychiatric phenotypes, neurocognitive endophenotypes, and regional brain circuitry activities. Second was to test whether the domain approach guided by RDoC could be an alternative avenue to elucidate “gene–brain–behavior” relationships of the *CADM1* gene by a series of domain-specific probes on different intermediate phenotypes within one single study, instead of relying on traditional diagnostic phenotypes alone. In other words, as an exploratory and hypotheses-generating study, we sought to explore the possible “gene–brain–behavior” relationship between *CADM1* genetic polymorphism, brain circuitries activities, and executive tasks performance relevant to ADHD. We postulated that the “domain-based” analyses for complex relationships could yield findings with finer specificity than conventional phenotypes.

## Materials and Methods

### Participants

In total, 2,003 individuals (1,040 children with ADHD and 963 healthy controls) participated in the current study. All ADHD probands were recruited from the child psychiatric clinics at Peking University Sixth Hospital/Institute of Mental Health. Psychiatric diagnoses of ADHD and comorbidities were assessed and classified according to Diagnostic and Statistical Manual of Mental Disorders, 4th Edition criteria (American Psychiatric Association, 2013) by trained child psychiatrists using the Chinese-translated version of the Clinical Diagnostic Interview Scale (Barkley, 1998; Liu and Guan, 2011) for a semi-structured interview with probands and their parents together. Comorbidities as captured by the Clinical Diagnostic Interview Scale included: oppositional defiance disorders, conductive disorder, tic disorder, learning disorder, obsessive–compulsive disorder, specific phobias, anxiety, depression, and bipolar disorder.

The inclusion criteria for ADHD probands included: 1) having a Diagnostic and Statistical Manual of Mental Disorders, 4th Edition ADHD diagnosis; 2) aged between 6 and 16 years; 3) with a full-scale IQ ≥ 70, as measured by the Chinese version of Wechsler Intelligence Scale for Children (Gong and Cai, 1993); and 4) both biological parents were of Chinese Han descent. Exclusion criteria included: a psychiatric history of schizophrenia, affective disorder, pervasive development disorders (or autism spectrum disorders), major physical or metabolic disorders, and neurological disorders. Healthy control subjects were of Chinese Han descent (include both adults and children) and recruited from three sources: students from local elementary schools; healthy blood donors attending the Blood Center of Peking University First Hospital; and healthy volunteers attending The Institute of Mental Health (Beijing) for research. Among recruited controls, those found to have ADHD, other major psychiatric disorders, family history of psychosis, severe physical diseases, and substance abuse were excluded. Adult control participants were screened by the ADHD Rating Scale (DuPaul et al., 1998) and self-report. Child control participants were screened i) for low IQ by the Chinese version of Wechsler Intelligence Scale for Children and, ii) for psychopathologies, by parent-rated ADHD Rating Scale (DuPaul et al., 1998), Conners’ Parent Rating Scale (Conners et al., 1998), and Achenbach’s Child Behavior Cheek-list (Tseng et al., 1988).

A subsample of 35 ADHD cases and 56 healthy controls (aged between 8 and 16 years) was recruited for the nested imaging genetic study. To control for potential confounders relevant to this imaging genetic study, more stringent inclusion and exclusion criteria were introduced. Additional exclusion criteria were post-traumatic stress disorder, enuresis, and encopresis [captured by Schedule for Affective Disorders and Schizophrenia for School-Age Children-Present and Lifetime version (Chinese-translated version) (Kaufman et al., 1997)]. Participants were also excluded for having conditions contraindicated for undergoing MRI procedures; these included: having metal implants (including nonremovable dentures) and claustrophobia. More stringent inclusion criteria were applied for controlling potential artifacts and confounders relevant to neuroimaging studies; these included: 1) right hand dominant; 2) no history of severe head injury or brain trauma (leading to loss of consciousness or coma); 3) full-scale IQ ≥ 80; and 4) ADHD medication effects. Only drug-naïve participants in the ADHD group were recruited.

This project was approved by the Ethics Committee of Peking University Sixth Hospital/Institute of Mental Health. Written informed consents were sought and obtained from parents for the child participants and from adult participants.

### Genotyping and Single Nucleotide Polymorphism Selection

Blood samples of both cases and controls were collected and genotyped using the Affymetrix6.0 array at CapitalBio Ltd. (Beijing) according to the standard Affymetrix protocol. Samples of cases and controls were added in equal proportion to each chip to avoid batch effects. The Affymetrix 6.0 array included 96 single nucleotide polymorphisms (SNP) probes of *CADM1* gene. The final set of 10-tag SNPs (**Table 1**) was selected based on two criteria: 1) common SNP sites according with the Hardy–Weinberg equilibrium and had a minor allele frequency above 5%; 2) using the confidence interval (CI) method of haplotype analysis software HaploView (ver4.2) to identify linkage disequilibrium, then tag SNPs yielded with the threshold setting of *r* ^2^ > 0.80 were included for the subsequent analysis.

**Table 1:** Basic information of 10 analyzed single nucleotide polymorphisms of *CADM1*.

Gene symbol	NCBI SNP reference	Allele	Public location (GRCh38)	SNP type	HWE	Call rate (%)	MAF
CADM1	rs11605461	A/G	chr.11-115182291	Intron	0.717	99.9	0.203
CADM1	rs11215407	A/G	chr.11-115194573	Intron	0.533	100	0.387
CADM1	rs7482812	C/T	chr.11-115212452	Intron	0.747	100	0.372
CADM1	rs10790068	C/T	chr.11-115230847	Intron	0.897	100	0.309
CADM1	rs10458969	A/G	chr.11-15232473	Intron	0.152	99.9	0.275
CADM1	rs17118125	A/G	chr.11-115244012	Intron	0.345	100	0.203
CADM1	rs10891819	G/T	chr.11-115266527	Intron	0.872	99.7	0.291
CADM1	rs10502204	C/T	chr.11-115274185	Intron	0.576	100	0.323
CADM1	rs7952231	G/T	chr.11-115337279	Intron	0.403	97.4	0.27
CADM1	rs220860	A/C	chr.11-115423345	Intron	0.759	99.9	0.203

HWE, Hardy–Weinberg equilibrium; MAF, minor allele frequency.

### Executive Function Measures

Executive function measures were ascertained in the subsample of ADHD and child control.

*Stroop Color–Word Interference test*: The test included four conditions: i) color naming (condition 1, e.g., name patches of color); ii) word reading (condition 2, e.g., read the rows of words printed in black ink); iii) color inhibition (condition 3, e.g., read the word *red* printed in *green* ink); iv) word inhibition (condition 4, e.g., name the color of the *green* ink rather than the word *red*). The “color interference time” denotes the average time (in seconds) taken to complete each trial of condition 3 subtracted by condition 2, and “word interference time” denotes the average time (in seconds) taken to complete each trial of condition 4 subtracted by condition 1. In this study, color and word interference time scores are analyzed to represent “inhibition” of executive function (Shuai et al., 2011).

*Rey–Osterrieth complex figure test (RCFT)*: RCFT evaluates visuospatial construction ability, visual working memory, and organizational skills. Participants were asked first to inspect and then copy the RCFT figure. After 30 s (Immediate Recall Condition) and 20 min (Delayed Recall Condition), they were asked to recall and reproduce the figure from memory without any visual cues or prompt. Both immediate and delayed scores were rated according to i) the structure recalled (structure score, 0–6 for 3 items) and ii) detailed accuracy reproduced (detail score, 0–36 for 18 items). “Forgotten” scores (for “structure” and for “detail”) were generated by subtracting respective “delay” scores from “immediate” scores. This yielded two set of scores: “structure forgotten score” and “detail forgotten score,” indicating the information that was lost during the 20-min interval. These discrepancy scores were analyzed in this study to represent “visual working memory” of executive function (Shuai et al., 2011).

*Trail making test*: This test was used to assess “set-shifting.” It includes two parts: i) number sequencing trail making; ii) number–letter switching trail making. The time taken to complete each part was recorded. The “set-shifting time” was represented by the time discrepancy taken for part 2 subtracted by the time taken to complete part 1 (Shuai et al., 2011).

### Magnetic Resonance Imaging Acquisition

All MRI images were acquired on the 3-T Siemens Tim Trio MRI scanner (Siemens, Erlangen, Germany) with a standard 12-channel head coil in the Imaging Center for Brain Research, Beijing Normal University. Participants while awake were instructed to remain still and relaxed with eyes closed during the 30-min period of rs-fMRI scanning, and a head strap and foam pads were used to minimize head movements. Functional images were acquired using an echo-planar imaging sequence with the following parameters: 33 axial slices, thickness/skip = 3.5/0.7 mm, repetition time = 2,000 ms, echo time = 30 ms, flip angle = 90°, matrix = 64 × 64, field of view = 200 × 200 mm, and 240 volumes. T1-weighted anatomical images were acquired with the following parameters: 128 slices, slice thickness = 1.33 mm, repetition time = 2,530 ms, inversion time (TI) = 1,100 ms, echo time = 3.39 ms, flip angle = 7°, matrix = 256 × 256, and field of view = 256 × 256 mm.

### Data Preprocessing

Analysis of the rs-fMRI data was performed using Data Processing & Analysis for Brain Imaging (DPABI) 4.4 toolbox (DPABI_V3.1) (Yan et al., 2016). The preprocessing included the following procedures: 1) removal of the first 10 volumes; 2) slice-timing correction; 3) head-motion correction; all the subjects head motion were lower than our criteria of 3 mm and 3°; 4) coregistration of T1 image to the functional image, and T1 image was segmented into gray matter, white matter, and cerebrospinal fluid by using the “new segment” method; 5) spatial normalization of segmented T1 image to standard Montreal Neurological Institute (MNI) space using “Dartel”; then, the functional data were normalized to the MNI space (resampled voxel size = 3 × 3 × 3 mm); 6) the Gaussian kernel full width at half-maximum was 6 mm3; 7) removal of linear trends; and temporal band pass filtering (0.01–0.08 Hz) were conducted; 8) regression of head motion effects with the Friston-24 parameter model, white matter, cerebrospinal fluid, and global signal.

### Mean Amplitude of Low-Frequency Fluctuation Calculation

ALFF, regional homogeneity, and degree centrality are the three most commonly used methods for “voxel-wise whole-brain” analysis in rs-fMRI (Zang et al., 2015). The intra-scanner reliability (i.e., test–retest reliability) of mALFF has been identified as having higher reliability than regional homogeneity and degree centrality (Zhao et al., 2018). In this study, mALFF was used as the matrix to represent resting state brain neural circuit activity.

The power spectrum was obtained by fast Fourier transform of the pretreated time courses, and the averaged square root across a frequency band of 0.01–0.08 Hz was calculated as ALFF (Zang et al., 2007). ALFF of each voxel was divided by the global mean ALFF for standardization purpose, and mALFF was obtained as a parameter for further statistical comparison and analysis.

### Statistical Analysis

#### Demographic and Clinical Characteristics

Demographic and clinical characteristics were compared between ADHD and control groups. *Chi*-square tests were applied for the categorical variable (sex), and independent sample *t*-tests were applied for continuous variables (age and IQ scores).

#### Gene–Behavior Association Analysis

First, the allelic and genotypic distributions under additive model of SNPs between “ADHD-whole” group and controls were compared using chi-square tests. Once the allelic and/or additive model difference reached nominal significance (*P* < 0.05), further genotypic comparisons under recessive and dominant models were conducted. Furthermore, we repeated the earlier analyses by stratifying the full ADHD sample (i.e. the "ADHD-whole" sample)into “ADHD-comorbid” and “ADHD-alone” subsamples to account for potential heterogeneity related to comorbidities within the ADHD phenotype, given the concerns raised about such effects on genetic association in the literature (Robinson et al., 2014). To correct for multiple comparisons, Bonferroni correction was performed setting significant *P*-value at 0.0008 (i.e., 0.05/10/2/3; with 10 representing the number of SNPs analyzed, 2 representing the allelic and genotypic models, and 3 representing the three analyzed phenotypes including “ADHD-whole,” “ADHD-alone,” and “ADHD-comorbid”). In addition, further logistic regression analyses were conducted to adjust the potential confounding effects with age, sex, and 10 principal components derived from the multidimensional scaling procedure for the Affymetrix 6.0 genotyping data (Yang et al., 2013) as covariates.

To minimize the artifacts introduced by multiple testing, only significant SNPs identified with positive associations in the case–control analysis were included in the general linear model that examines the association between genotypes and executive function measures. General linear model (multiple linear regression model with more than one dependent variable) was performed using SPSS. The executive function measures (as represented by the scores for “Stroop color interference time,” “Stroop word interference time,” “RCFT structure forgotten score,” “RCFT detail forgotten score,” and “TMT set-shifting time”) were entered as dependent variables in the general linear model, while genotypes were entered as independent variables, with age, sex, IQ, and ADHD diagnoses set as covariates. Whenever genetic main effects were detected, *post hoc* analyses were then reconducted separately in the ADHD and control groups.

#### Imaging Genetic Analysis

Statistical analyses of mALFF were performed in DPABI (Yan et al., 2016). A mixed effect analysis was conducted in DPABI to determine whether there were any significant regional mALFF differences between genotypes and phenotype groups—ADHD and control groups. To control for multiple testing, AlphaSim correction was applied. By using AlphaSim correction, the significance threshold was set at *P* < 0.05 (a combination threshold of voxel level at *P* < 0.01 and a cluster size estimated by AlphaSim, with the kernel of smoothness recalculated based on four-dimensional residual).

#### Correlations of Genotype-Modulated Regional Mean Amplitude of Low-Frequency Fluctuations With Executive Function and Mediation Analyses

The correlations between regional mALFF alteration and executive functions (EF) indexes were conducted in those brain regions using Pearson correlation. Sex, age, IQ, and ADHD diagnoses were controlled as covariates.

If the correlation reached significance, moderation and mediation effects were evaluated using the PROCESS macro of SPSS (Hayes, 2017). First, moderation was assessed by model 1, in which the interaction effect of the W (the moderator, regional mALFF) and X (genotype) on Y (EF indexes) was computed. If there no moderation effect was detected, mediation was then assessed by model 4, in which the indirect effects of the X on Y through M (the mediator, regional mALFF) were evaluated for effect size and significance (Hayes and Rockwood, 2017). We used bootstrapping with 5,000 samples. The effects of sex, age, IQ, and ADHD cases were controlled as covariates. In the moderation analyses, the variables were mean-centered before the interactions were modeled. Sobel test of mediation (Sobel, 1982) was applied to determine whether regional mALFF significantly mediated the relations between rs10891819 genotype and executive function measures.

#### Expression Quantitative Trait Loci Analysis

To explore the potential biological functions of the SNPs identified in our first analysis, we further examined the patterns of expression quantitative trait loci (eQTL) based on the data from the UK Brain Expression Cohort (http://www.braineac.org).

## Results

### Demographic Data

The demographic and clinical characteristics of participants for both the association study and nested imaging genetic study are summarized in **Table 2** and **Supplementary Table 1**. Sex ratio and IQ scores differed between “ADHD-whole” and control groups, with male preponderance and lower IQ scores detected in the ADHD group. In the sample of the imaging genetic study, the mean age of the control group was higher (all *P*s < 0.05).

**Table 2:** Demographic and Clinical Characteristics of subjects recruited.

	Gene-behavior association study				Imaging genetic study			
	ADHD (n = 1,040)	Control (n = 963)	χ*^2^/t*	*P*	ADHD (n = 35)	Control (n = 56)	χ*^2^/t*	*P*
Male(%)	876 (84.2%)	607 (63)	115.9	<0.001	32 (91.4%)	25 (44.6%)	20.1	<0.001
Age [Mean (SD)]	9.2 (2.5)	15.0 (8.9)	19.0	<0.001	10.0 (1.7)	9.8 (1.8)	0.6	0.547
IQ [Mean (SD)]	103.9 (14.7)	112.7 (14.1)	9.3	<0.001	106.2 (14.9)	115.6 (12.7)	3.2	0.002
ADHD Subtype (%)								
ADHD-I	360 (34.6%)	–			14 (40%)	–		
ADHD-C	680 (65.4%)	–			21 (60%)	–		
Comorbidities (%)								
ADHD-comorbid	746 (71.7%)	–			26 (74.3%)	–		
ADHD-alone	295 (28.3%)	–			9 (25.7%)	–		

ADHD, attention-deficit/hyperactivity disorder; ADHD-I, ADHD inattentive subtype; ADHD-C, ADHD combined subtype; ADHD-alone, ADHD subjects without any comorbidity; ADHD-comorbid, ADHD with assessed comorbidities; IQ, intelligence quotient; SD, standard deviation.

### Gene–Behavior Association Analyses

No differences in allelic and genotypic distribution of any SNP examined were found in the “ADHD-whole” sample or “ADHD comorbid” subsamples when compared with controls (**Supplementary Table 2**). In the “ADHD-alone” subsample, the genotypic distribution of rs10891819 was different from the controls at the nominal levels of significance in both additive model (*P* = 0.008) and the recessive model with TT genotype as protective [odds ratio = 0.48 (95% CI, 0.27–0.85), *P* = 0.012] (**Table 3**). Further adjustment for covariates (age and sex and the 10 principal components from the multidimensional scaling procedure) yielded similar results (**Table 3**). All the earlier results could not survive Bonferroni corrections. Quanto 1.2.4 was used to evaluate the statistical power of our sample. The power estimate yielded 73% at alpha of 0.05, based on the respective values in sample size, prevalence, allele frequency, and relative risk (ADHD cases = 295; healthy controls = 963; prevalence = 0.05; allele frequency = 0.29; inherent mode = recessive; relative risk of alleles = 0.48). We then repeated the same analysis in the “ADHD-whole” sample, “ADHD-comorbid,” and “ADHD-alone” subsamples specifically using the child-only control subsample (i.e., excluding the adult controls for a more stringent validation); the results did not differ substantially (data not shown).

**Table 3:** Allelic and Genotypic Analysis in ADHD-alone (n = 295) and Controls (n = 963).

SNP	A1	A2	Allelic comparison			Genotypic comparison					
			A1/A2 in case: control	OR (95% CI)	*P*	Additive		Dominant		Recessive	
						A1A1/A1A2/A2A2 in case: control	*P*	OR (95% CI)	*P*	OR (95% CI)	*P*
rs11605461	**A**	G	456/126: 1536/390	0.92 (0.73–1.15)	0.464	174/108/9: 614/308/41	0.213				
rs11215407	**A**	G	380/208: 1156/770	1.22 (1.00–1.48)	0.045	127/126/41: 347/462/154	0.085				
rs7482812	C	**T**	213/373: 719/1207	0.96 (0.79–1.16)	0.666	41/131/121: 130/459/374	0.670				
rs10790068	**C**	T	404/184: 1321/605	1.00 (0.82–1.23)	0.956	138/128/28: 460/401/102	0.789				
rs10458969	**A**	G	165/421: 511/1411	1.08 (0.88–1.33)	0.453	23/119/151: 64/383/514	0.722				
rs17118125	**A**	G	473/115: 1531/395	1.06 (0.84–1.34)	0.616	187/99/89: 609/313/41	0.481				
rs10891819	G	**T**	418/168: 1353/565	1.04 (0.85–1.27)	0.713	139/140/14: 485/383/91	0.008	1.12 (0.87–1.46)	0.384	0.48 (0.27–0.85)	0.012
rs10502204	C	**T**	200/388: 628/1298	1.07 (0.88–1.30)	0.525	32/136/126: 96/436/431	0.812				
rs7952231	G	**T**	142/428: 500/1380	0.92 (0.74–1.14)	0.423	17/108/160: 68/364/508	0.697				
rs220860	A	**C**	469/119: 1545/377	0.96 (0.76–1.21)	0.740	191/87/16: 617/311/33	0.236				
**Logistic regression with covariates** **^a^**											
rs10891819	G	**T**	418/168: 1353/565	1.02 (0.80–1.29)	0.895	139/140/14: 485/383/91	0.042	1.19 (0.88–1.62)	0.258	0.55 (0.29–1.04)	0.067

ADHD-alone, ADHD subjects without any comorbidity; OR: odd ratios; 95% CI: 95% confidence interval; the ancestry alleles were bolded.

a Age, sex, and the 10 principal components from the multidimensional scaling procedure as covariates.

For the “gene-EF” analyses, performances on all executive function measures were poorer in the ADHD group compared with controls. However, no genotypic main effect of rs10891819 was detected for the “ADHD-whole,” “ADHD-alone,” or “ADHD-comorbid” grouping (**Supplementary Table 3**) (all *P*s > 0.05).

### Imaging Genetic Study

The genotypic distributions of rs10891819 in the nested study were: 19, 12, and 4 carriers of GG, GT, and TT genotypes in the “ADHD-whole” group (n = 35) and 37, 13, and 6 carriers in control group (n = 56). However, when stratified by comorbidities, no TT genotype carriers were found in the “ADHD-alone” subgroup. Subsequently, the GT and TT genotypes were combined to form the “T-allele carrier” group for imaging genetic analyses. Further details on sample characteristics were given in **Supplementary Table 4**. No genotypic effect was found on any EF parameters (**Supplementary Table 5**) (all *P*s > 0.05).

**Figure 1:**
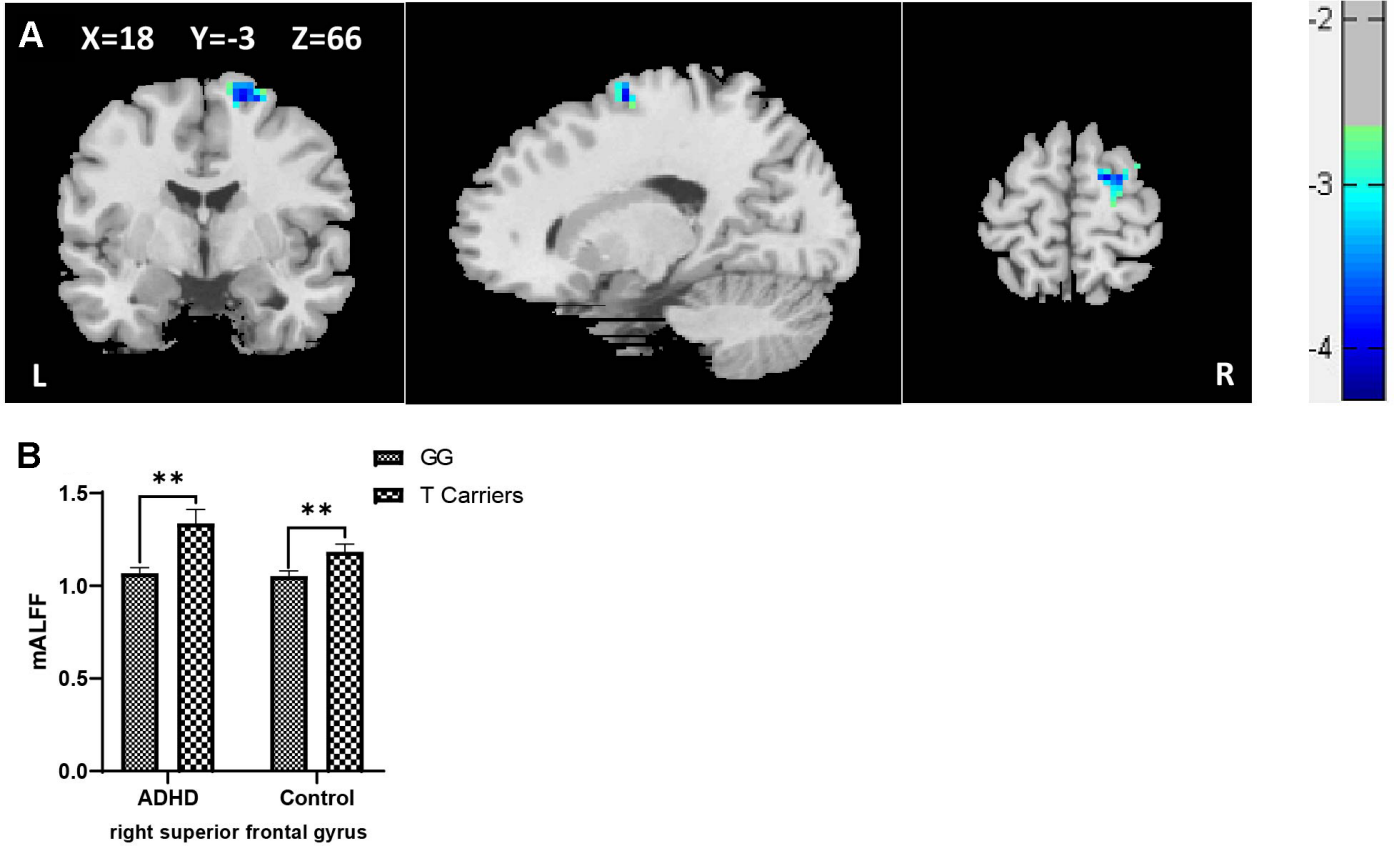
**(A)** Regional ALFF differences between GG carriers and T allele carriers of rs10891819 in right superior frontal gyrus (GG < T allele carriers). **(B)** Contrasts of regional mALFF values were shown by main effect of rs10891819 genotype. X, Y, Z shows the MNI coordinates of the peak F/t value. **P < 0.01.

When controls and ADHD cases were analyzed as a combined group, a main genotypic effect of rs10891819 on the brain activity was detected. Specifically, significantly higher levels of mALFF were detected in the right superior frontal gyrus (rSFG) for T-allele carriers when compared with GG carriers (peak *t* = 3.85, corrected *P* < 0.05, also see **Table 4A**, **Figure 1A**). *Post hoc* analyses stratifying ADHD and control participants into two separate groups detected the same pattern: T-allele carriers showed significantly higher levels of mALFF than GG carriers in the ADHD group [(1.34 ± 0.30) versus (1.07 ± 0.13), *P* = 0.001] and in the control group [(1.18 ± 0.18) versus (1.05 ± 0.16), *P* = 0.002] (also see **Supplementary Table 6**, **Figure 1B**). These results survived AlphaSim correction.

**Table 4A:** Effect of genotype on mean amplitude of low-frequency fluctuation in combined samples of ADHD-whole and control.

Effect	Cluster	Brain regions(AAL)	Number of voxels	Peak MNI coordinates			Peak *F/t*-value
				X	Y	Z	
Genotype							
GT/TT > GG	1	Frontal_Sup_R	43	18	-3	66	3.85

ADHD, attention-deficit/hyperactivity disorder; AAL, anatomical automatic labeling; age, sex, and IQ as covariates. Threshold: voxel p < 0.01, cluster P < 0.05 after AlphaSim correction.

When the ADHD cases were stratified into “ADHD-alone” and “ADHD-comorbid” subgroups and then combined with the participants from control group, respectively (for sufficient statistical power for comparison), the genotypic effect of rs10891819 remained significant in the rSFG region, with higher levels of mALFF shown in T-allele carriers compared with GG carriers (**Supplementary Table 6**) (all *P*s < 0.05).

### Correlational, Moderation, and Mediation Analyses for Genotype, Regional mALFF, and Executive Function Measures

**Figure 2:**
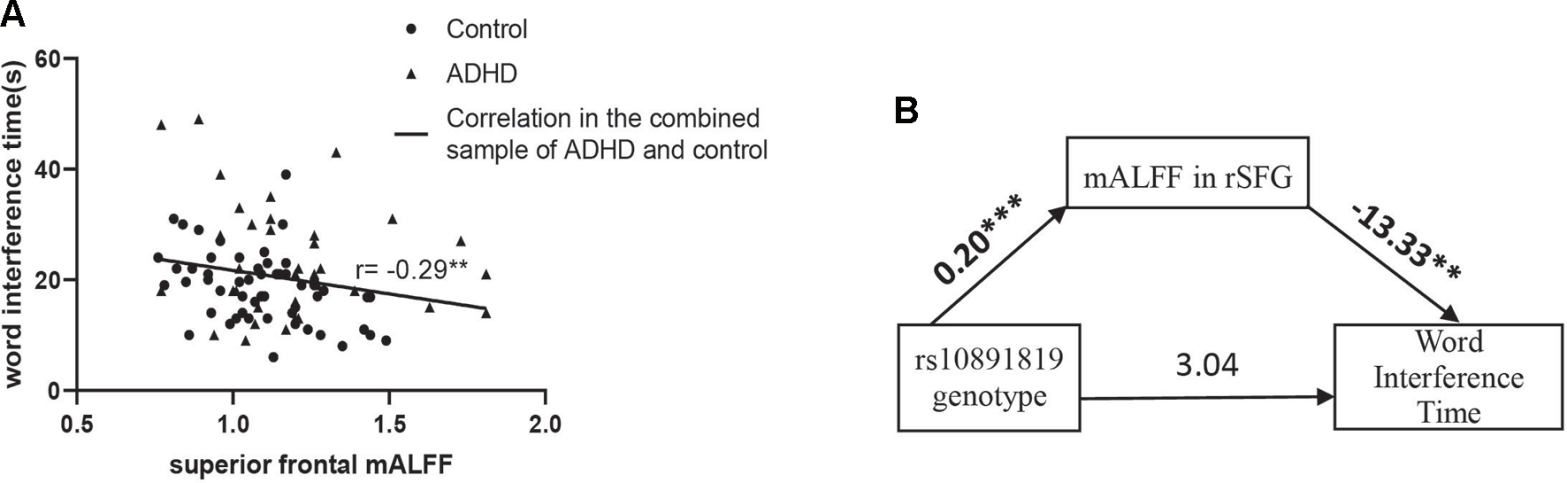
**(A)** “Word interference time” of Stroop task (i.e., low values indicating higher inhibition function) was negatively correlated with mALFF in right superior frontal gyrus where the main effect of genotype detected in the combined sample (n = 91) of ADHD participants (n = 35) and controls (n = 56). **(B)** Shows the full-mediation model of mALFF on the relationship between genotype and word interference time. Numbers above the arrow lines indicate the unstandardized effects of variables in start points on those in end points. rSFG: right superior frontal gyrus. **P < 0.01, ***P < 0.001.

Correlation analyses of regional mALFF (in the rSFG) and executive function measures were conducted in the combined sample of ADHD participants and controls. The results showed a negative correlation between mALFF in the rSFG and “word interference time” in the STROOP test (*r* = -0.29, *P* = 0.006, **Figure 2A**), indicating higher mALFF levels correlated with better performance in this inhibition task. However, the correlation between mALFF and other remaining executive measures was not detected (*P*s > 0.05, **Table 4B**). When stratifying ADHD and control participants into two separate groups, we could only detect in the control group the negative correlation between mALFF in the rSFG and “word interference time” in STROOP test (r = -0.41, P = 0.003).

**Table 4B:** Correlation between mean amplitude of low-frequency fluctuation in right superior frontal gyrus and executive measures in combined samples of ADHD-whole and control.

EF performance	mALFF	
	*r*	*P* *^a^*
Structure Recall Error score	-0.05	0.640
Detail Recall Error score	0.05	0.635
Set-shifting time	0.06	0.616
Color Interference time	0.16	0.140
Word Interference time	-0.29	0.006

a Adjusted with age, sex, IQ, and ADHD diagnoses.

Using PROCESS, the moderation effect of mALFF in rSFG (moderator) on the relationship between genotype (X) and word interference time (Y) was evaluated. The level of mALFF (moderator) was significantly associated with word interference time, but there was no significant mALFF*genotype interaction in relation to word interference time (**Supplementary Table 7**).

The mediation effect was then examined to evaluate the three-way relationship between mALFF in the rSFG (mediator), genotype (X), and word interference time (Y). In the mediation model, the path from genotype to mALFF was significant [B = 0.20 (SE = 0.04), 95% CI = 0.12 to 0.27, *P* = 2.10 × 10^-6^], and the path from mALFF to word interference time was significant [B = -13.33 (SE = 4.72), 95% CI = -3.39 to -22.73, *P* = 0.006], but the path from genotype to word interference time did not reach statistical significance. The bias-corrected bootstrap 95% CI indicated that the indirect path through mALFF was significant [B = -2.61 (SE = 1.07), 95% CI = -0.48 to -4.72, **Supplementary Table 7**]. An indirect-only subtype of mediation was detected (Zhao et al., 2010): Sobel test for mediation effect was significant (Sobel z = -2.47, *P* = 0.009) offering support that mALFF mediated the path between genotype and word interference time (**Figure 2B**).

### Expression Quantitative Trait Loci Analyses for rs10891819

**Figure 3:**
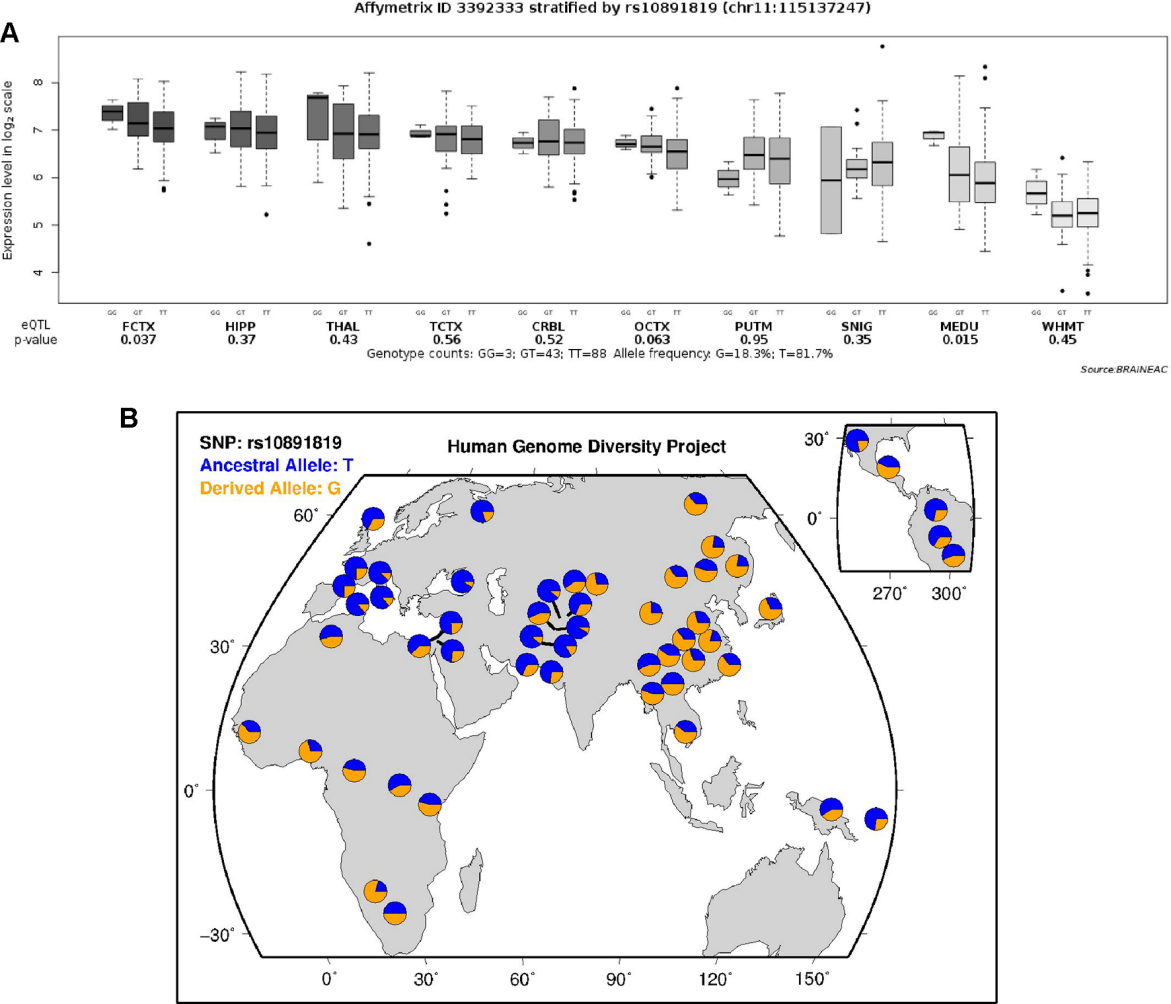
**(A)** Expression quantitative trait loci analysis of rs10891819 on CADM1 transcriptional expression in human brain. FCTX, frontal cortex; HIPP, hippocampus; THAL, thalamus; TCTX, temporal cortex; CRBL, cerebellar cortex; OCTX, occipital cortex (specifically primary visual cortex); PUTM, putamen; SNIG, substantia nigra; MEDU, medulla (specifically inferior olivary nucleus); WHMT, intralobular white matter. **(B)** Worldwide diversity of rs10891819 allele frequencies in Human Genome Diversity Project (http://genome.ucsc.edu/trash/hgc/hgdpGeo_rs10891819.png).

According to the data extracted from online resource from the UK Brain Expression Cohort (of Caucasian participants), the minor G allele of the SNP rs10891819 was associated with higher *CADM1* expression level (P-value = 0.037, **Figure 3A**). This pattern was different from our sample of Chinese Han participants, in whom T variant was the minor allele (**Figure 3B**).

## Discussion

Our study examined the association of *CADM1* gene in relation to ADHD psychiatric phenotypes, neurocognitive endophenotypes, and regional brain circuitry activities. There are four key findings.

The first key finding was a marginal significant genotypic effect of rs10891819 detected only in the “ADHD-alone” subgroup, with TT genotype as protective, though the association did not survive Bonferroni correction. Second, in the nested imaging genetic study, rs10891819 genotype was significantly associated with altered spontaneous regional brain activities during rs-fMRI, in the rSFG region. More specifically, the mALFF activities in the T-allele carriers were consistently higher than GG carriers in both ADHD and control groups. Third, endophenotypic correlation analyses detected a significant negative correlation between “word interference time” in Stroop and mALFF activity in the rSFG, that is: higher spontaneous regional brain activities in the rSFG were correlated with better performance in inhibition task (as indexed by shorter “word interference time”). Fourth, our mediation analysis confirmed a significant three-way effect (supported by a significant Sobel test for “indirect-only mediation”) from “gene” to “brain activity” to “inhibition task”—potentially representing a “gene–brain–behavior” relationship. The significant indirect effects involved two paths: from rs10891819 genotype (T-allele carriers) to brain activation in the rSFG (higher activities) and from rSFG to Stroop inhibition task (better performance).

In other words, we only detected a protective effect of *CADM1* genotype and its association with higher brain activation in the context of better performance in inhibition task. These two strands of findings are consistent with each other, suggestive that the detected *CADM1* genotypic effects confer better cognitive function and therefore protection, rather than elevating the risks of impaired cognitive processes or phenotypic expression of ADHD. Our preliminary findings could also indicate that *CADM1* genotypes may not directly elevate the risk of ADHD expression and therefore would be consistent with our predictions derived from ADHD GWAS, which did not detect genome-wide significant associations with the “disorder phenotype.”

Our second aim was to test whether the domain approach guided by RDoC could be an alternative avenue to elucidate “gene–brain–behavior” relationships of the *CADM1* gene by a series of domain-specific probes on different intermediate phenotypes within one single study, instead of relying on traditional diagnostic phenotypes alone. The SNP rs10891819 showed marginal association (TT genotype as protective) in the “ADHD-alone” subsample. The detected association was not found in the “ADHD-comorbid” or the whole (unstratified) sample. This could represent a spurious chance finding (Liu et al., 2015) or a weak genetic signal partially obscured by other unmeasured confounders. Inevitably, this preliminary finding needs to be replicated by future studies with larger sample size. Within the remits of our study, we then applied the domain approach guided by RDoC and interrogated this weak genetic signal further using a nested imaging genetic study. Through iterations along the domains posited by RDoC, other significant findings were uncovered in brain activities in relation to genotype and cognitive intermediate phenotype. Finally, a significant mediation model emerged: delineating the paths from “T-allelic carrier genotype” to “higher brain activation in the rSFG” and from “rSFG” to “better performance in inhibition task.” The findings are congruent with the theoretical and biological plausibility that the detected “better performance in inhibition” is in line with our other findings, such as “higher brain activity in the PFC” involved top–down control as well as the “detected protective effect” against ADHD expression. Given the small sample size in our imaging genetic study and multiple testing conducted (without surviving Bonferroni correction), our findings should be interpreted with caution and regarded as exploratory. As a hypotheses-generating study, our findings provided preliminary support for the merits of domain-informed approach based on RDoC framework in exploring potential “gene–brain–behavior” relationships within the context of *CADM1* gene. Future studies with larger samples may specifically test these hypotheses generated by our exploratory findings. It is particularly striking that the mediation effect on “gene–brain–endophenotype” relationship was detected independent of the clinical diagnostic phenotypes (i.e., in ADHD and/or control groups). If such findings were replicated, our findings may offer support for the RDoC conceptual framework that privileges brain circuitries (in relation to genes and endophenotypes) over the clinical phenotypes as the primary anchor for investigation.

Our findings were in line with the suggestion derived from a recent study that cell adhesion pathway could be an etiological candidate for ADHD (Lima et al., 2016), but the association is unlikely to be a linear one or conforming to the conventional bivariate model of risk and disease. *CADM1* gene encodes cell adhesion molecule 1, which influences a wide range of neural functions, including neuronal development, myelination, synaptic formation, plasticity, and integrity of neuronal networks (Lima et al., 2016). Genes involved in neuronal migration, growth, morphology, synaptic plasticity, and cell adhesion have been implicated by GWAS in ADHD (Zayats et al., 2015; Lima et al., 2016).

Interestingly, rs10891819 is located in intron 9 of *CADM1* gene, a region with uncertain but putative function of influencing expression of CADM1 protein molecule. As shown from the expression quantitative trait loci analyses, the minor G allele (in Caucasian samples) was associated with a higher *CADM1* expression level. However, the reverse pattern of minor allele of rs10891819 was observed in our Chinese Han participants (T as minor allele) (**Figure 3B**); one possible explanation is that the T allele in Chinese Han and G allele in Caucasian subjects might confer same postulated function, relevant to the expression of ADHD symptoms—given the putative protective function bestowed by a higher expression of *CADM1* and positive downstream influences on higher prefrontal neural activities and better inhibitory control. However, there are no available expression data in Hans population to support this interpretation, and we could only infer higher cognitive performance observed in our findings attributable to better functions of CADM1 protein molecule. If our findings were replicated, future study may be needed to evaluate the transcriptional functions of *CADM1* polymorphism and elucidate more fully their functional roles in Chinese Han participants. In addition, the possible mechanism for the involvement of *CADM1* in ADHD has also been considered within dopaminergic functions in a recent review (Kitagishi et al., 2015). Evidently, dopamine transporter (DAT) is a key molecule in psychopharmacological treatment of ADHD, pivotal in i) regulating the DA level within synaptic cleft and ii) maintaining presynaptic DA function through synthesis and storage. However, the regulatory functions of DAT are dependent on protein kinase (PKA) and protein kinase B (AKT), which are activated by phosphatidylinositol-3-hydroxykinase (PI3K). By recruiting PI3K to the membrane surface, CADM1 molecule plays a putatively crucial role in affecting the upstream signaling pathways of DAT and, consequently, in the pathogenesis of ADHD (Kitagishi et al., 2015). Therefore, the potential roles of *CADM1* gene involved in the expression of ADHD symptoms are complex and likely implicated at multiple levels: including at the level of specific pathway (e.g., cell adhesion) and at the level of pathway–pathway interaction (e.g., “cell adhesion pathway” intersecting with “monoaminergic pathway”). Moreover, the genotypic effect of *CADM1* on rSFG and subsequent relationship with inhibition function reported by our research might be the consequence of CADM1*DAT1 gene–gene interaction. ADHD is likely a disorder involving multiple causal genes of small effects and interactions. To elucidate this possibility, future study with *DAT1* and other genotypes can unpack more fully the effects and theoretical implications of CADM1*DAT1 and other gene–gene interactions.

Several limitations need to be considered. First, most of the findings did not survive correction for multiple testing. Our study is an exploratory study examining the genetic effects of *CADM1* gene on ADHD, and it should be regarded as a hypothesis-generating study. Second, the scope was limited by the small sample size, especially after stratification by comorbidities status. Our preliminary findings should be treated with caution and needed to be replicated in other samples. More specifically, Caucasian samples may show an opposite effect; given G-allele is the minor allele conferring higher *CADM1* expression in Caucasian population. Future replication studies should therefore be vigilant of potential divergent functional effects of a given minor allele on cellular, brain, functional, and behavioral expression. Third, the participants in our controls recruited in the gene–behavior association analyses included both adults and children. Genotypes do not change with age, and healthy adult samples without childhood history of psychiatric disorders can be used as controls. Further validation analyses in children-only samples could overcome this limitation. In addition, if some adults failed to recall or disclose childhood disorders accurately, contamination of the controls by ADHD cases would reduce the statistical power of the sample, biasing the results toward the null hypothesis rather than leading to spurious positive findings. Fourth, there were multiple testing and comparison in our study. Our significant findings could be spurious and arose by chance. However, the directions of significant findings converged meaningfully in line with theoretical and biological plausibility, regarding the protective effect of the rs10891819 genotype, higher PFC activation, better cognitive function, and their mediating relationship. It remained likely that a weak genetic signal initially detected by candidate gene association approach was amplified through subsequent domain iterations as guided by RDoC approach. Fifth, we could only detect the effect of an “indirect-only mediation” (Zhao et al., 2010). Zhao et al. (2010) provided an extensive review on different subtypes of mediation model. Our final mediation conformed to the “indirect-only mediation” subtype. It is possible that the long chain of intermediates between *CADM1* gene and inhibition endophenotype has diluted the direct effect to the extent that our small sample could not detect a significant association between gene and inhibition. Alternatively, the “brain-activation” phenotype embodies two unrelated or lowly correlated variances, and each one of them correlates with *CADM1* gene and inhibition independently—as a result, only “brain” correlates with both. Future studies with a larger sample adequately powered (based on our detected effect sizes) may be able to provide a fuller explanation. Furthermore, mediation and moderation analyses by future studies could be utilized in a gene–environment interaction model justified by a plausible biological theory (van der Meer et al., 2015). Our findings should be regarded as hypothesis generating and should only serve as a stimulus for future research.

In conclusion, our study offers preliminary evidence to support the roles of *CADM1* function in relation to prefrontal brain activities, inhibitory executive function, and ADHD phenotype, implicating a potential “gene–brain–behavior” relationship of the *CADM1* gene. Our preliminary findings derived from this hypotheses-generating study also provided support for the merits of applying the domain-informed approach based on RDoC framework in ADHD research. Future studies with larger samples may specifically test these hypotheses generated by our exploratory findings.

## Data Availability

The raw data supporting the conclusions of this manuscript will be made available by the corresponding author on reasonable request, without undue reservation, to any qualified researcher.

## Ethics Statement

This project was approved by the Ethics Committee of Peking University Sixth Hospital/Institute of Mental Health. Written informed consents were sought and obtained from parents for the child participants and from adult participants.

## Author Contributions

JJ, QG, LL, YW and QQ contributed conception and design of the study. HL, JJ, and QG organized the database. JJ, LL, WC, YW and QQ performed the statistical analysis, interpreted the results, and wrote sections of the manuscript. All authors contributed to manuscript revision and read and approved the submitted version.

## Funding

This work was supported by the National Science Foundation of China (81571340), the National Key Basic Research Program of China (973 program 2015CB856405), Beijing Municipal Science & Technology Commission (No.Z161100000516032), the National Natural Science Foundation of China (81873802, 81641163, 81761148026), Beijing Natural Science Foundation (7172245), and the Capital Foundation of Medical Developments (CFMD:2016-2-4113).

## Conflict of Interest Statement

The authors declare that the research was conducted in the absence of any commercial or financial relationships that could be construed as a potential conflict of interest.
