# Supplementary material for: The Implicated Roles of Cell Adhesion Molecule 1 (CADM1) Gene and Altered Prefrontal Neuronal Activity in Attention-Deficit/Hyperactivity Disorder: A “Gene–Brain–Behavior Relationship”?
Source: Front Genet. 2019 Sep 26;10:882. doi: 10.3389/fgene.2019.00882 (PMC6775240; doi:10.3389/fgene.2019.00882)
Supplement: Supplementary file 1 [file Table_1.docx]

**Supplementary materials**

**Supplementary Table 1. Demographic and Clinical Characteristics of subjects recruited**

|  | **ADHD** | | | |  | **Control** | | | |
| --- | --- | --- | --- | --- | --- | --- | --- | --- | --- |
|  | **Genetic (n=1040)** | **Imaging**  **genetic (n=35)** | ***χ^2^/t*** | ***P*** |  | **Genetic (n=963)** | **Imaging genetic (n=56)** | ***χ^2^/t*** | ***P*** |
| **Male(%)** | 876 (84.2%) | 32 (91.4%) | 1.34 | 0.248 |  | 607 (63%) | 25 (44.6%) | 1.42 | 0.234 |
| **Age** **[Mean(SD)]** | 9.2(2.5) | 10.0(1.7) | 1.88 | 0.061 |  | 15.0(8.9) | 9.8(1.8) | 4.37 | **<0.001** |
| **IQ [Mean(SD)]** | 103.9(14.7) | 106.2(14.9) | 0.91 | 0.363 |  | 112.7(14.1) | 115.6 (12.7) | 1.50 | 0.133 |
| **ADHD Subtype (%)** |  |  |  |  |  |  |  |  |  |
| ADHD-I | 360(34.6%) | 14(40%) | 0.43 | 0.511 |  | - | - |  |  |
| ADHD-C | 680(65.4%) | 21(60%) |  |  |  | - | - |  |  |
| **Comorbidities (%)** |  |  |  |  |  |  |  |  |  |
| ADHD-comorbid | 746(71.7) | 26(74.3) | 0.12 | 0.735 |  | - | - |  |  |
| ADHD-alone | 295(28.3) | 9(25.7) |  |  |  | - | - |  |  |

Note. ADHD, attention deficit hyperactivity/disorder; ADHD-I, ADHD inattentive subtype; ADHD-C, ADHD combined subtype; ADHD-alone, ADHD subjects without any comorbidity; ADHD-comorbid, ADHD with assessed comorbidities;IQ, intelligence quotient; SD, standard deviation.

**Supplementary Table 2.** **Allelic and Genotypic Analysis in 1040 ‘ADHD-whole’ (746 ‘ADHD-comorbid’) Samples and 963 Controls**

| **SNP** | **A1** | **A2** | **Allele** | | |  | **Genotype** | |  |
| --- | --- | --- | --- | --- | --- | --- | --- | --- | --- |
|  |  |  | **A1/A2 in Case : Control** | **OR**  **(95%CI)** | ***P*** |  | **Additive Model** | | |
|  |  |  |  |  |  |  | **A1A1/A1A2/A2A2 in Case: Control** | ***P*** | |
| **ADHD-whole** | | |  |  |  |  |  |  | |
| rs11605461 | **A** | G | 1653/421:  1536/390 | 1.00  (0.85-1.16) | 0.969 |  | 654/345/38:  614/308/41 | 0.692 | |
| rs11215407 | **A** | G | 1302/778: 1156/770 | 1.12  (0.98-1.27) | 0.094 |  | 400/502/138:  347/462/154 | 0.188 | |
| rs7482812 | C | **T** | 770/1308: 719/1207 | 0.99  (0.87-1.12) | 0.857 |  | 143/484/412:  130/459/374 | 0.889 | |
| rs10790068 | **C** | T | 1447/633: 1321/605 | 1.05  (0.92-1.20) | 0.503 |  | 498/154/91:  460/401/102 | 0.348 | |
| rs10458969 | **A** | G | 591/1487: 511/1411 | 1.10  (0.96-1.26) | 0.190 |  | 75/441/523:  64/383/514 | 0.369 | |
| rs17118125 | **A** | G | 1661/419: 1531/395 | 1.02  (0.88-1.19) | 0.774 |  | 670/321/49:  609/313/41 | 0.683 | |
| rs10891819 | G | **T** | 1479/597: 1353/565 | 1.04  (0.90-1.19) | 0.626 |  | 517/445/76:  485/383/91 | 0.143 | |
| rs10502204 | C | **T** | 666/1414: 628/1298 | 0.97  (0.85-1.11) | 0.691 |  | 107/452/481:  96/436/431 | 0.717 | |
| rs7952231 | G | **T** | 552/1470: 500/1380 | 1.04  (0.90-1.19) | 0.620 |  | 66/420/525:  68/364/508 | 0.421 | |
| rs220860 | A | **C** | 1643/437: 1545/377 | 0.92  (0.79-1.07) | 0.273 |  | 656/331/53:  617/311/33 | 0.187 | |
| **ADHD-comorbid** | | | | |  |  |  |  | |
| rs11605461 | **A** | G | 1197/295:  1536/390 | 1.03  (0.87-1.22) | 0.730 |  | 480/237/29:  614/308/41 | 0.919 | |
| rs11215407 | **A** | G | 922/570:  1156/770 | 1.08  (0.94-1.24) | 0.292 |  | 273/376/97:  347/462/154 | 0.212 | |
| rs7482812 | C | **T** | 557/935:  719/1207 | 1.00  (0.87-1.15) | 0.999 |  | 102/353/291:  130/459/374 | 0.989 | |
| rs10790068 | **C** | T | 1043/449:  1321/605 | 1.06  (0.92-1.23) | 0.408 |  | 360/323/63:  460/401/102 | 0.317 | |
| rs10458969 | **A** | G | 426/1066:  511/1411 | 1.10  (0.95-1.28) | 0.202 |  | 52/322/372:  64/383/514 | 0.327 | |
| rs17118125 | **A** | G | 1188/304:  1531/395 | 1.01  (0.85-1.19) | 0.924 |  | 483/222/41:  609/313/41 | 0.286 | |
| rs10891819 | G | **T** | 1061/429:  1353/565 | 1.03  (0.89-1.20) | 0.671 |  | 378/305/62:  485/383/91 | 0.690 | |
| rs10502204 | C | **T** | 466/1026:  628/1298 | 0.94  (0.81-1.09) | 0.393 |  | 75/316/355:  96/436/431 | 0.461 | |
| rs7952231 | G | **T** | 410/1042:  500/1380 | 1.09  (0.93-1.27) | 0.292 |  | 49/312/365:  68/364/508 | 0.215 | |
| rs220860 | A | **C** | 1174/318:  1545/377 | 0.90  (0.76-1.07) | 0.221 |  | 465/244/37:  617/311/33 | 0.268 | |

Note. OR: odd ratios; 95% CI: 95% confidence interval; ADHD-whole, the whole ADHD sample; ADHD-comorbid, ADHD with assessed comorbidities. The ancestry alleles were bolded.

**Supplementary Table 3. Association between EF performance and rs10891819 genotypes in ‘ADHD-whole’ (n=497), ‘ADHD-alone’ (n=140), ‘ADHD-comorbid’ and control (n=116) ^a^**

| **EF performance** | **Group** | **Mean±SD** | | | ***F_disease_***  ***(p)*** | ***F_genotype_***  ***(p)*** |
| --- | --- | --- | --- | --- | --- | --- |
|  |  | GG | GT | TT |  |  |
| **ADHD-whole** |  |  |  |  |  |  |
| Structure Forgotten score | Control | 0.20±0.61 | 0.13±0.41 | 0.02±0.21 | 5.89  (0.016) | 0.50  (0.607) |
|  | ADHD | 0.37±0.83 | 0.33±0.74 | 0.43±1.09 |  |  |
| Detail Forgotten score | Control | 1.20±1.77 | 0.76±1.07 | 0.18±0.41 | 6.27  (0.013) | 1.04  (0.355) |
|  | ADHD | 1.40±2.09 | 1.23±1.70 | 1.65±3.04 |  |  |
| Set-shifting time | Control | 96.22±90.57 | 88.04±65.67 | 70.73±49.31 | 18.84  (1.7×10^-5^) | 1.92  (0.148) |
|  | ADHD | 143.11±106.96 | 156.02±105.44 | 123.81±82.77 |  |  |
| Color Interference time | Control | 4.88±3.69 | 5.02±4.23 | 3.82±2.40 | 16.74  (4.9×10^-5^) | 0.03  (0.975) |
|  | ADHD | 8.28±8.20 | 8.57±9.46 | 8.41±9.56[ |  |  |
| Word Interference time | Control | 22.40±9.95 | 23.62±9.24 | 20.82±12.54 | 22.96  (2.0×10^-6^) | 0.16  (0.852) |
|  | ADHD | 29.68±16.57 | 31.32±17.65 | 29.49±15.05 |  |  |
| **ADHD-alone** |  |  |  |  |  |  |
| Structure Forgotten score | Control | 0.20±0.61 | 0.13±0.41 | 0.02±0.21 | 4.71  (0.031) | 0.31  (0.736) |
|  | ADHD | 0.41±0.90 | 0.36±0.64 | 0.40±0.89 |  |  |
| Detail Forgotten score | Control | 1.20±1.77 | 0.76±1.07 | 0.18±0.41 | 6.10  (0.014) | 0.44  (0.643) |
|  | ADHD | 1.56±2.42 | 1.45±2.03 | 3.00±5.66 |  |  |
| Set-shifting time | Control | 96.22±90.57 | 88.04±65.67 | 70.73±49.31 | 10.61  (0.001) | 1.71  (0.183) |
|  | ADHD | 157.69±104.73 | 157.78±114.34 | 114.40±51.53 |  |  |
| Color Interference time | Control | 4.88±3.69 | 5.02±4.23 | 3.82±2.40 | 10.52  (0.001) | 0.15  (0.861) |
|  | ADHD | 8.78±8.51 | 9.01±10.75 | 8.00±3.67 |  |  |
| Word Interference time | Control | 22.40±9.95 | 23.62±9.24 | 20.82±12.54 | 15.91  (8.8×10^-5^) | 1.69  (0.187) |
|  | ADHD | 30.81±19.54 | 35.66±16.51 | 36.40±14.80 |  |  |
| **ADHD-comorbid** | | |  |  |  |  |
| Structure Forgotten score | Control | 0.20±0.61 | 0.13±0.41 | 0.02±0.21 | 5.36  (0.021) | 0.49  (0.611) |
|  | ADHD | 0.36±0.61 | 0.31±0.78 | 0.35±0.83 |  |  |
| Detail Forgotten score | Control | 1.20±1.77 | 0.76±1.07 | 0.18±0.41 | 4.87  (0.028) | 1.35  (0.252) |
|  | ADHD | 1.35±1.97 | 1.12±1.51 | 1.44±2.50 |  |  |
| Set-shifting time | Control | 96.22±90.57 | 88.04±65.67 | 70.73±49.31 | 20.41  (8.0×10^-6^) | 1.22  (0.296) |
|  | ADHD | 137.87±107.54 | 155.15±101.20 | 143.32±103.69 |  |  |
| Color Interference time | Control | 4.88±3.69 | 5.02±4.23 | 3.82±2.40 | 17.85  (2.9×10^-5^) | 0.09  (0.917) |
|  | ADHD | 8.11±8.10 | 8.35±8.79 | 8.47±10.22 |  |  |
| Word Interference time | Control | 22.40±9.95 | 23.62±9.24 | 20.82±12.54 | 21.81  (4.0×10^-6^) | 0.34  (0.715) |
|  | ADHD | 29.27±9.95 | 29.18±17.85 | 28.41±15.03 |  |  |

Note. ADHD-whole, the whole ADHD sample; ADHD-alone, ADHD subjects without assessed comorbidities; ADHD-comorbid, ADHD with assessed comorbidities

**^a^** Adjusted with age, sex and IQ

**Supplementary Table 4. Demographic Information of Subjects in Imaging Genetics Study**

|  |  | **rs10891819_GG** | **rs10891819_GT/TT** | ***F/x^2^*** | ***P*** |
| --- | --- | --- | --- | --- | --- |
| **Age** |  |  |  |  |  |
|  | ADHD-whole | 10.7(2.0) | 10.3(1.5) | 0.323 | 0.574 |
|  | ADHD-alone | 9.8(1.6) | 10.0(2.0) | 0.137 | 0.895 |
|  | ADHD-comorbid | 10.3(2.0) | 9.9(1.4) | 0.68 | 0.505 |
|  | Control | 9.8(1.9) | 9.7(1.5) | 0.063 | 0.803 |
|  | Total | 10.1(1.9) | 10(1.5) | 0.106 | 0.746 |
| **Sex (male/female)** |  |  |  |  |  |
|  | ADHD-whole | 19/0 | 13/3 | 1.87 | 0.086 |
|  | ADHD-alone | 6/0 | 3/0 | ---- | ---- |
|  | ADHD-comorbid | 13/0 | 10/3 | 3.39 | 0.066 |
|  | Control | 17/20 | 8/11 | 0.075 | 0.784 |
|  | Total | 36/20 | 21/14 | 0.169 | 0.681 |
| **IQ score** |  |  |  |  |  |
|  | ADHD-whole | 106.6(11.1) | 105.8(18.8) | 0.022 | 0.882 |
|  | ADHD-alone | 106.2(4.6) | 100.0(14.7) | 0.708 | 0.547 |
|  | ADHD-comorbid | 106.8(13.3) | 107.2(19.9) | 0.06 | 0.954 |
|  | Control | 115(13.8) | 116.7(11.5) | 0.199 | 0.658 |
|  | Total | 112.1(13.4) | 111.7(16) | 0.012 | 0.914 |

Note. ADHD-whole, the whole ADHD sample; ADHD-alone, ADHD subjects without assessed comorbidities; ADHD-comorbid, ADHD with assessed comorbidities

**Supplementary Table 5. Association between EF performance and rs10891819 genotypes in imaging genetic subgroup ^a^**

| **EF performance** | **Group** | **Mean±SD** | | ***F_disease_***  ***(p)*** | ***F_genotype_***  ***(p)*** |
| --- | --- | --- | --- | --- | --- |
|  |  | GG | GT&TT |  |  |
| Structure Forgotten score | Control | 0.18±0.50 | 0.07±0.27 | 0.11  (0.739) | 0.93  (0.339) |
|  | ADHD | 0.29±0.61 | 0.11±0.33 |  |  |
| Detail Forgotten score | Control | 1.20±1.72 | 0.26±0.57 | 0.80  (0.347) | 2.62  (0.112) |
|  | ADHD | 1.00±1.18 | 0.82±1.28 |  |  |
| Set-shifting time | Control | 111.25±108.03 | 77.03±55.89 | 0.14  (0.708) | 0.00  (0.972) |
|  | ADHD | 107.29±54.41 | 144.31±100.43 |  |  |
| Color Interference time | Control | 3.67±2.98 | 5.22±5.04 | 0.18  (0.671) | 3.49  (0.067) |
|  | ADHD | 3.79±4.39 | 6.63±3.68 |  |  |
| Word Interference time | Control | 20.23±7.07 | 17.29±6.33 | 1.79  (0.187) | 0.01  (0.924) |
|  | ADHD | 21.00±10.55 | 24.36±8.08 |  |  |

**^a^** Adjusted with age, sex and IQ

**Supplementary Table 6. Association between mALFF in rSFG and rs10891819 genotypes in ‘ADHD-whole’ (n=35), ‘ADHD-alone’ (n=9), ‘ADHD-comorbid’ (n=26) and control (n=56) ^a^**

| **Group** | **mALFF (Mean±SD)** | | ***F*** | ***P*** |
| --- | --- | --- | --- | --- |
|  | GG | GT&TT |  |  |
| **ADHD-whole**  **and control** | 1.06±0.15 | 1.25±0.25 | 25.93 | 2.1×10^-6^ |
| **ADHD-alone**  **and control** | 1.06±0.16 | 1.20±0.22 | 11.88 | 0.001 |
| **ADHD-comorbid**  **and control** | 1.06±0.16 | 1.25±0.24 | 23.7 | 5.9×10^-6^ |

Note. ADHD-whole, the whole ADHD sample; ADHD-alone, ADHD subjects without assessed comorbidities; ADHD-comorbid, ADHD with assessed comorbidities

**^a^** Adjusted with age, sex, IQ and ADHD diagnoses

Supplementary Table 7. Moderation and Mediationof mALFF on the relationship between genotype andWord Interference timein combined samples of ADHD-whole and control^a^

| **Variables** | **Moderation on Word Interference time (Y)** | | | | |  | **mALFF in rSFG (M)** | | | | |  | **Word Interference time (Y)** | | | | |
| --- | --- | --- | --- | --- | --- | --- | --- | --- | --- | --- | --- | --- | --- | --- | --- | --- | --- |
|  | **β** | **SE** | **t** | **95%CI^b^** | |  | **β** | **SE** | **t** | **95%CI^b^** | |  | **β** | **SE** | **t** | **95%CI^b^** | |
|  |  |  |  | **LLCI** | **ULCI** |  |  |  |  | **LLCI** | **ULCI** |  |  |  |  | **LLCI** | **ULCI** |
| **constant** | 31.91 | 10.13 | 3.15** | 11.75 | 52.06 |  | 0.41 | 0.23 | 1.82 | -0.04 | 0.87 |  | 42.95 | 10.12 | 4.25*** | 22.83 | 63.07 |
| **genotype (X)** | 3.09 | 1.94 | 1.60 | -0.76 | 6.93 |  | 0.20 | 0.04 | 5.09*** | 0.12 | 0.27 |  | 3.04 | 1.91 | 1.59 | -0.76 | 6.84 |
| **mALFF (M/W)** | -14.03 | 5.65 | -2.49* | -25.26 | -2.8 |  |  |  |  |  |  |  | -13.33 | 4.73 | -2.82** | -22.73 | -3.93 |
| **genotype*mALFF** | 2.15 | 9.40 | 0.23 | -16.55 | 20.85 |  |  |  |  |  |  |  |  |  |  |  |  |
| **R^2^** | 0.24 | | | | |  | 0.38 | | | | |  | 0.24 | | | | |
| **F** | 3.66** | | | | |  | 10.57*** | | | | |  | 4.31** | | | | |
| **Mediation (Indirect effect of X on Y)** |  | | | | |  |  | | | | |  | **β** | **BootSE** | | **95%CI^b^** | |
|  |  | | | | |  |  | | | | |  |  |  |  | **LLCI** | **LLCI** |
|  |  | | | | |  |  | | | | |  | -2.61 | 1.07 | | -4.83 | -0.53 |

Note.X: independent variable; Y: dependent variable; W: moderator; M: mediator; 95% CI: 95% confidence interval; LLCI: lower limit confidence interval; ULCI: upper limit confidence interval. *P<0.05, **P<0.01, ***p<0.001

**^a^** Adjusted with age, sex, IQ and ADHD diagnoses;**^b^** Effects are significant when the upper and lower bound of the bias corrected 95% confidence intervals (CI) does not contain zero.
